# Deep hybrid model for maternal health risk classification in pregnancy: synergy of ANN and random forest

**DOI:** 10.3389/frai.2023.1213436

**Published:** 2023-07-05

**Authors:** Taofeeq Oluwatosin Togunwa, Abdulhammed Opeyemi Babatunde, Khalil-ur-Rahman Abdullah

**Affiliations:** ^1^Department of Medicine and Surgery, Faculty of Clinical Sciences, College of Medicine, University of Ibadan, Ibadan, Oyo, Nigeria; ^2^College Research and Innovation Hub, University College Hospital, Ibadan, Oyo, Nigeria; ^3^MyBelle Digital Maternal and Child Health Organisation, Ibadan, Nigeria; ^4^Public Health Interest Group Africa (PHIGA), Lagos, Nigeria; ^5^Faculty of Clinical Sciences, College of Health Sciences, University of Ilorin, Ilorin, Nigeria; ^6^MCON Institute of Medical Research, Ilorin, Nigeria

**Keywords:** maternal health, artificial neural networks, random forest, deep learning, machine learning, artificial intelligence

## Abstract

**Introduction:**

Maternal health is a critical aspect of public health that affects the wellbeing of both mothers and infants. Despite medical advancements, maternal mortality rates remain high, particularly in developing countries. AI-based models provide new ways to analyze and interpret medical data, which can ultimately improve maternal and fetal health outcomes.

**Methods:**

This study proposes a deep hybrid model for maternal health risk classification in pregnancy, which utilizes the strengths of artificial neural networks (ANN) and random forest (RF) algorithms. The proposed model combines the two algorithms to improve the accuracy and efficiency of risk classification in pregnant women. The dataset used in this study consists of features such as age, systolic and diastolic blood pressure, blood sugar, body temperature, and heart rate. The dataset is divided into training and testing sets, with 75% of the data used for training and 25% used for testing. The output of the ANN and RF classifier is considered, and a maximum probability voting system selects the output with the highest probability as the most correct.

**Results:**

Performance is evaluated using various metrics, such as accuracy, precision, recall, and F1 score. Results showed that the proposed model achieves 95% accuracy, 97% precision, 97% recall, and an F1 score of 0.97 on the testing dataset.

**Discussion:**

The deep hybrid model proposed in this study has the potential to improve the accuracy and efficiency of maternal health risk classification in pregnancy, leading to better health outcomes for pregnant women and their babies. Future research could explore the generalizability of this model to other populations, incorporate unstructured medical data, and evaluate its feasibility for clinical use.

## 1. Introduction

Maternal health is a crucial aspect of public health as it directly affects the wellbeing of both mother and child. Despite advances in medical science, maternal mortality remains a significant problem in many countries, particularly in developing nations (Olonade et al., 2019; Musarandega et al., 2021). Maternal and childhood mortality remains a major global health concern and a key indicator for the United Nations Sustainable Development Goal 3 (SDG 3). According to the World Health Organization (WHO), about 810 pregnant women and 6,700 newborns die every day (WHO, 2019, 2020). Although, most of these mortalities are preventable. The major causes of maternal mortality are preeclampsia/eclampsia, postpartum hemorrhage, sepsis, and delivery complications (Say et al., 2014; Sageer et al., 2019). Maternal Mortality is defined as the annual number of female deaths from any cause related to or aggravated by pregnancy or its management, excluding accidental or incidental causes, during pregnancy and childbirth or within 42 days of termination of pregnancy, irrespective of the duration and site of the pregnancy (WHO, 2017). In 2014, in anticipation of the launch of the SDGs, the WHO released the Targets and strategies for ending preventable maternal mortality (EPMM) which had a target of reducing the Global Maternal Mortality Rate (MMR) to <70% by 2030 (WHO, 2019). This highlights the WHO's commitment to improving maternal health worldwide.

Identifying high-risk pregnancies is a critical step in ensuring maternal and fetal health, but it can be a challenging task due to the complexity and variability of the factors involved. Maternal health risk during pregnancy refers to the likelihood of a woman experiencing adverse health outcomes during pregnancy or childbirth. Pregnancy can be graded into low, moderate, and high risk based on risk factors that have been shown to contribute to the occurrence of pregnancy complications (Al-Hindi et al., 2020). High-risk pregnancies can pose a risk of perinatal complications to the mother such as preeclampsia, gestational diabetes mellitus, and cesarean section delivery (Ben Naftali et al., 2018); or to the offspring such as prematurity, perinatal asphyxia, congenital anomalies and even cardiovascular abnormalities in adulthood (Majella et al., 2019; Yoon, 2021). These detrimental effects undoubtedly impose serious health risks on both the mother and the fetus. Risk factors for adverse maternal health outcomes during pregnancy can include a variety of factors such as age, pre-existing medical conditions, lifestyle factors, and socioeconomic status (Londero et al., 2019; Crear-Perry et al., 2021). Consequently, women who are from disadvantaged backgrounds or who lack access to quality healthcare services may be at higher risk of adverse maternal health outcomes. Identifying and managing maternal health risks during pregnancy is critical to maternal health care. In addition, pregnant women have continuously been excluded from most clinical trials, hence, data on the efficacy and safety of medications in pregnancy are often lacking (van der Graaf et al., 2018). Therefore, in order to give precedence to the research interest of pregnant women and enhance the development of secure and efficacious diagnostic and therapeutic tools, it is crucial to focus on the establishment of safe and non-invasive methodologies. Within this context, the utilization of cutting-edge technologies would offer an unparalleled approach, paving the way for innovative solutions in this domain.

Healthcare providers use a variety of tools and assessments to identify women who may be at high risk for adverse outcomes, including medical history, physical exams, and laboratory tests. A 39-item validated instrument was designed by the Alberta perinatal health program in 2020 to categorize pregnant women attending antenatal into high, moderate and low risk (Al-Hindi et al., 2020). The system assigned weighted values to each of these items and a calculated aggregate would determine the risk category of the pregnant women. Unfavorable pregnancy outcomes were higher among pregnant women categorized as high-risk, while low-risk pregnancy had the least morbidities. Hence, early identification of these risk factors, categorization of pregnant women and management of pregnancy based on risk-level can play a major role in improving outcomes and reducing maternal and newborn mortalities.

In recent years, artificial intelligence (AI) has emerged as a powerful tool in healthcare, offering new ways to analyze and interpret complex medical data. AI-based models have shown promising results in various clinical applications, including disease diagnosis, treatment planning, and patient monitoring. AI-based models can analyze vast amounts of health data, both structured and unstructured, to identify patients who may be at high risk for adverse outcomes. These models can help healthcare providers make more accurate and timely decisions; and when it refers to maternal health care in pregnancy, it can ultimately improve maternal and fetal health outcomes. AI offers novel approaches to prediction modeling, diagnosis as well as early detection of pregnancies at risk (Ramakrishnan et al., 2021). A study by Akazawa and Hashimoto (Akazawa and Hashimoto, 2022) evaluated the accuracy of AI in predicting preterm birth in pregnancy, in a bid to increase preparedness and reduce neonatal deaths. The common predictive values used were electrohysterogram images, the metabolic panels in amniotic fluid or maternal blood, obstetric ultrasound images of the cervix, and biological profiles of mothers. Also, AI has been used in predicting the onset of pregnancy conditions such as preeclampsia, and gestational diabetes mellitus, and in the management of diseases such as ectopic pregnancy (Abuelezz et al., 2022). Besides, there is a growing acceptability of the usage of AI in clinical settings among patients. In a recent study conducted by Armero, Gray (Armero et al., 2022), 69% of pregnant women surveyed agreed to AI usage in maternal healthcare delivery, although, level of education and knowledge about AI were major factors that influenced the women's acceptability.

Furthermore, a 2019 study applied five advanced AI algorithms (Neural network, SVM, Random forest, Adaboost, and Multi-Layer Perceptron) to predict gestational diabetes from collected clinical data (Shen et al., 2019). The Random forest algorithm was found to be the best with a sensitivity of prediction of over 70%, outperforming doctors who solely rely on the fasting plasma glucose criteria in low medical resource conditions. Accordingly, the use of AI algorithms, such as Random Forest, can lead to more accurate and earlier diagnosis of various maternal conditions in pregnancy, in this case gestational diabetes, particularly in low resource settings where access to medical professionals may be limited. This would result in improved treatment outcomes, reduced burden on medical professionals, and potential cost savings. However, there remains ethical considerations around algorithmic bias, transparency, and patient autonomy which must always be taken into consideration. In addition, a more recent study by Kilicarslan, Celik (Kilicarslan et al., 2021) used hybrid models; genetic algorithm and deep learning algorithms of Stacked Autoencoder (SAE) and Convolutional Neural Network (CNN) for the prediction of nutritional anemia, (iron deficiency anemia, B12 deficiency anemia, and folate deficiency anemia), and patients without anemia. The results showed that the proposed algorithm performs better than previous studies with a 98.50% accuracy.

The widespread adoption of various machine learning (ML) algorithms and deep learning (DL) frameworks in supervised classification tasks can be attributed to their various advantages, including high operability, the capacity to handle nonlinear problems effectively, and the ability to circumvent data distribution constraints (Hu et al., 2021). Artificial neural networks have emerged as robust algorithms capable of modeling complex nonlinear relationships between input and output variables, and their application in healthcare decision-making has been extensively documented (Shahid et al., 2019; Vanem et al., 2022).

Among the diverse ML classification techniques, the Random Forest Classifier has been shown to outperform individual classifiers with its high classification accuracy; similarly exhibiting shorter training times compared to Decision Trees and Support Vector Machines, making it a fast and efficient option (Parmar et al., 2019). Additionally, it has the inherent ability to handle noise effectively, thus mitigating the overfitting issue. These advantageous characteristics have led to the growing popularity of the Random Forest approach within the research community, particularly for classification tasks.

Notably, more sophisticated frameworks known as transformers have emerged in recent times, and can possibly be re-engineered for supervised classification tasks with the potential of better performance. However, transformers are often highly computationally expensive and very time-consuming to train (Brown et al., 2020). Thus, they are not always the most suitable option.

Currently, one limitation of AI models is the use of small dataset (Akazawa and Hashimoto, 2022). Also, while AI can pave the way for novel findings in maternal health outcomes, AI without societal acceptance and integration is insufficient for impacting clinical care (Ramakrishnan et al., 2021). Thus, integrating AI tools into the maternal clinical setting requires support from all: doctors, statisticians, communities, organizations, engineers, governments, and mothers. Essentially, by improving our understanding of maternal health risk and utilizing advanced technologies such as AI, we can work toward ensuring that all women have access to safe and high-quality maternal health care, regardless of their socioeconomic status or geographic location.

Hence, the objective of this study is to use a hybrid model that integrates the predictive capabilities of traditional machine learning (ML) and deep learning (DL) algorithms, utilizing the Random Forest Classifier and Artificial Neural Network respectively, to classify maternal health risks during pregnancy. This paper will discuss the challenges in maternal health risk classification, the potential of AI in addressing these challenges, and the development of a deep hybrid model for maternal health risk classification.

## 2. Materials and methods

### 2.1. Data description and operating environment

**Figure 1:**
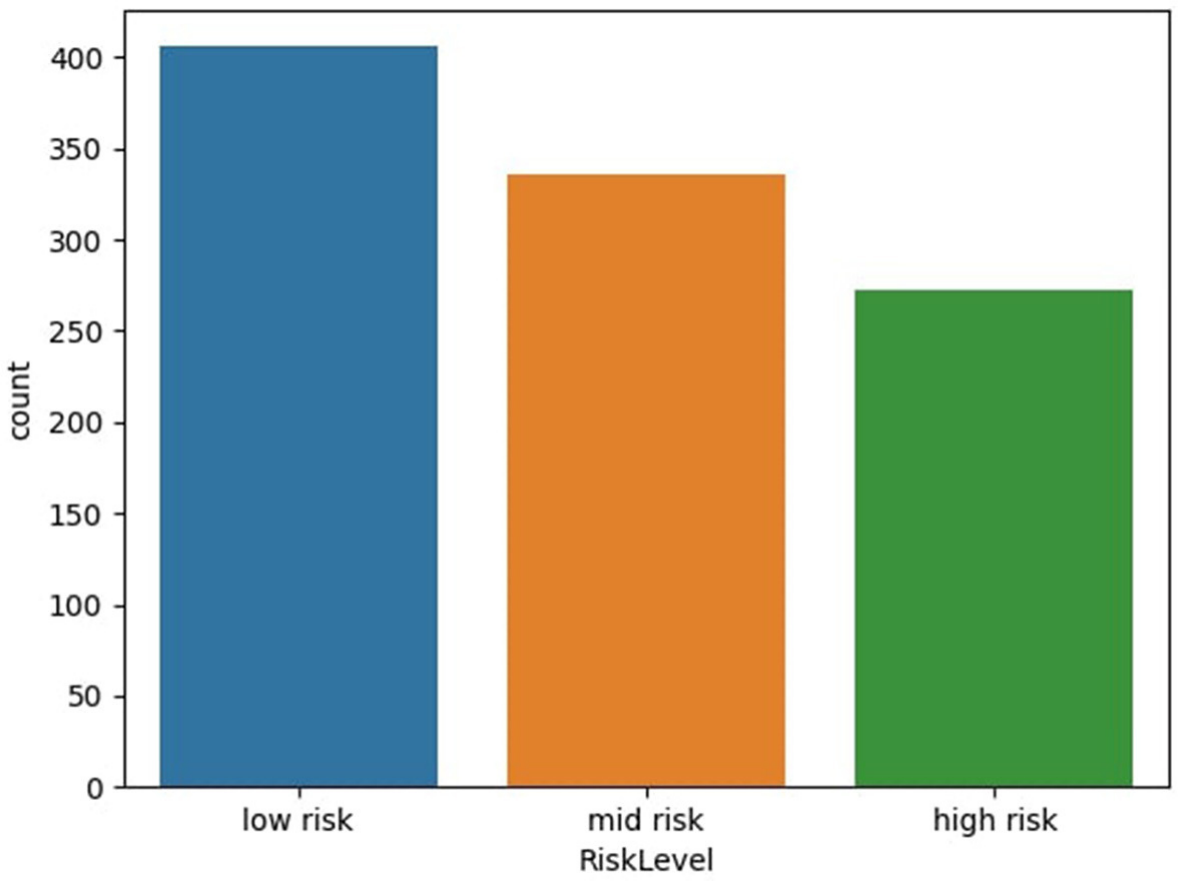
Distribution of dataset based on risk level. Figure shows the distribution of dataset based on risk level of subject—low risk, mid risk, and high risk.

The maternal health risk dataset utilized in this study was acquired from the University of California, Irvine (UCI) machine learning repository (Ahmed et al., 2020). The data was originally collected from different hospitals, community clinics and maternal health care centers from the rural areas of Bangladesh; through an IoT risk monitoring system. It was donated by Ahmed, Kashem (Ahmed et al., 2020) on 31st December, 2020. The dataset contained six predictive variables in pregnancy; Age (in years), Systolic Blood Pressure (in mmHg), Diastolic Blood Pressure (in mmHg), Blood Sugar (in mmol/L), Body Temperature (in degrees Fahrenheit) and Heart Rate (in beats per minute). All of which were numerical variables. A categorical target variable, Risk Level, was present in three possible classes; high risk, mid risk and low risk. These consequently totaled 7 variables in the whole dataset. There were 1,014 instances in the dataset; 406 samples of which were in the low risk class, 336 samples were in the mid risk class, and 272 samples were in the high risk class, as shown in Figure 1. Further feature statistics are shown in Table 1. There were no missing values in the dataset. All samples with all of their features were used in the model building and evaluation. All data manipulations, model building and evaluations were performed in the python programming language (version 3.8.3). Packages utilized include pycaret (2.3.10), tensorflow (2.11.0), numpy (1.20.3), pandas (1.5.2), sklearn (0.23.2).

**Table 1:** Description and statistical distribution of the dataset features.

	**Age**	**Systolic BP**	**Diastolic BP**	**Blood sugar**	**Body temperature**	**Heart rate**
Count	1014	1014	1014	1014	1014	1014
Mean	29.8718	13.1982	76.4605	8.7259	98.6650	74.3017
Std	13.4744	18.4039	13.8858	3.2935	1.3714	8.0887
Min	10.0000	70.0000	49.0000	6.0000	98.0000	7.0000
25%	19.0000	100.0000	65.0000	6.9000	98.0000	70.0000
50%	26.0000	120.0000	80.0000	8.0000	98.0000	76.0000
75%	39.0000	120.0000	90.0000	8.0000	98.0000	80.0000
Max	70.0000	160.0000	100.0000	19.0000	103.0000	90.0000

Table shows the statistical analysis of the age, systolic BP, diastolic BP, blood sugar, body temperature and heart rate variables.

### 2.2. Data pre-processing and analysis

**Figure 2:**
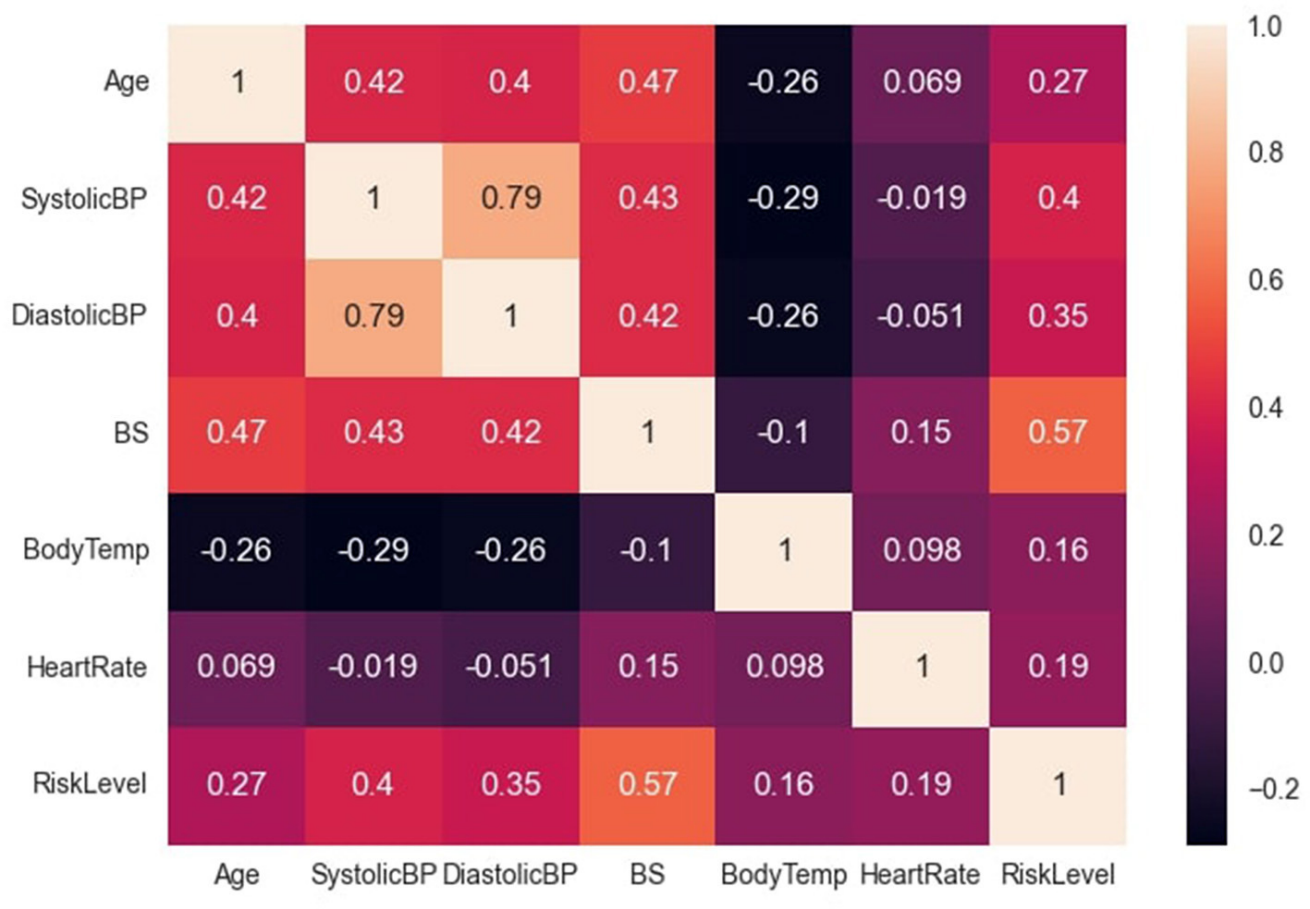
Correlation between dataset variables. Figure shows the correlation between the dataset variables.

The data file was saved in the comma separated value (csv) format, and this facilitated easy reading into the python script. The major preprocessing step carried out was coding the target (categorical) variables into numerical variables to facilitate computation. Low-risk, mid-risk and high-risk classes were coded as 0, 1, and 2 respectively. In addition, the previous data statistics indicated that the heart rate variable had a minimum value of 7, which is not biologically plausible and may have resulted from imputation error. Consequently, two data instances with this outlier value were identified (both recorded as low-risk) and re-imputed with the mode value of 70, before further analysis and modeling. Subsequently, the correlation between variables was examined, as depicted in Figure 2. The variable with the highest correlation to the risk level was blood sugar, while the variable with the lowest correlation was body temperature.

**Figure 3:**
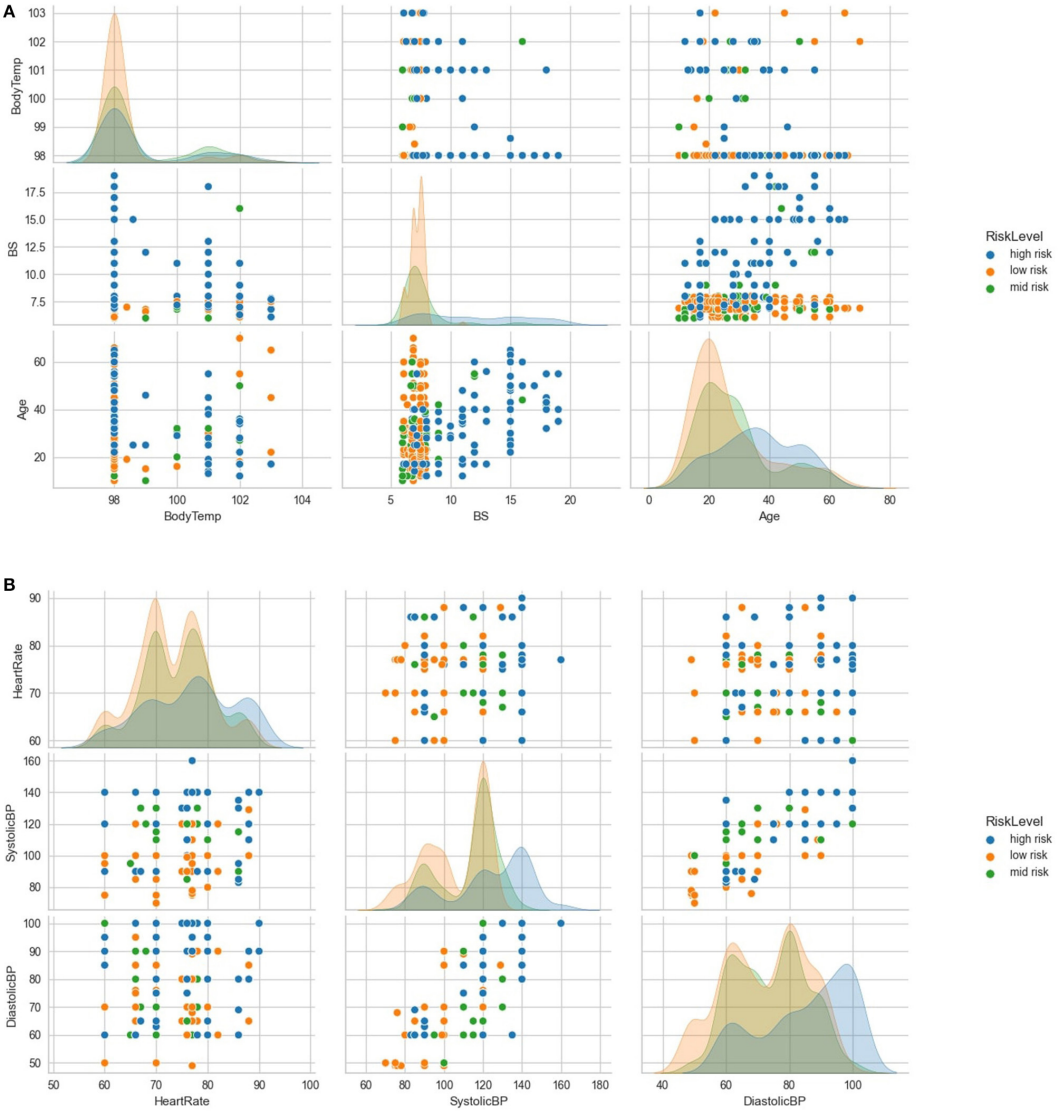
Further exploratory analysis of the variables. **(A)** shows further exploratory analysis of relationships and distributions between age, blood sugar, body temperature and risk levels. **(B)** shows further exploratory analysis of relationships and distributions between heart rate, systolic BP, diastolic BP, and risk levels.

Further exploratory analysis, as illustrated in Figure 3A, revealed several relationships between variables. Subjects over 50 years old with blood sugar levels above 15 mmol/L had a high-risk density. Additionally, even if their body temperature was within normal limits, subjects with blood sugar above 15 mmol/L were more likely to be classified as high-risk. Conversely, subjects with optimal blood sugar levels were likely to be classified as low-risk, even with increasing age. The general distribution showed a high low-risk density with low maternal age, peaking at 20 years; mid-risk as maternal age increased, and a high high-risk density with advanced maternal age, peaking at 35 years. The findings from the exploratory analysis suggest that certain variables, such as blood sugar levels and age, are associated with an increased risk of adverse health outcomes.

Further analysis, as demonstrated in Figure 3B, indicated that subjects with systolic blood pressure of 140 mmHg and above and diastolic blood pressure of 90 mmHg and above were more likely to be classified as high-risk. However, most subjects with optimal heart rates (between 70 and 80 beats per minute) and low systolic and diastolic blood pressure levels were classified as low-risk.

Furthermore, the data was randomly divided into training and testing sets, with 75% and 25% of the data, respectively. To facilitate data analysis, the Standard Scaler was applied to scale the data (Pedregosa et al., 2011), with the aim of approximating the standard normal distribution of the individual features. To compute the standard score “z” of a sample “x”, the following formula is used:


$$\begin{array}{l} z=\;\left(x\;-\;u\right)/s \end{array}$$

Here, “u” represents the mean of the training samples, and “s” represents the standard deviation of the training samples.

Subsequently, the training data was fitted and transformed using this scaling tool, while the testing data was transformed only.

### 2.3. Model architecture

#### 2.3.1. Artificial neural network

The ANN architecture used in this study had an input layer with six neurons, two hidden layers with five and four neurons respectively and an output layer with three neurons. The input layer and the hidden layers utilized the Rectified Linear Unit (ReLU) activation (Agarap, 2018), and the output layer used a softmax activation function (Gao and Pavel, 2017). The connection weights between the neurons were adjusted using an extended version of the stochastic gradient algorithm, known as the “Adam Optimizer” (Kingma and Ba, 2014). The loss function employed for parameter estimation during model training was categorical cross-entropy.

#### 2.3.2. Machine learning algorithm

The predictive abilities of various commonly used machine learning algorithms were initially examined- including Random Forest (RF) Classifier, Extra Trees Classifier, Light Gradient Boosting Machine, Decision Tree Classifier, Gradient Boosting Classifier, K Neighbors Classifier, Linear Discriminant Analysis, Ridge Classifier, Quadratic Discriminant Analysis, Ada Boost Classifier, Logistic Regression, Naive Bayes, Support Vector Machines—Linear Kernel and Dummy Classifier. We compared these different ML algorithms using the accuracy and the F1 score. The results are summarized in Table 2. The algorithm that performed best according to these metrics was the Random Forest Classifier. The architecture of the Random Forest comprised of 147 estimators with the gini criterion. This ensemble-based classifier was subsequently selected for further training.

**Table 2:** Comparison of different ML algorithms.

	**Model**	**Accuracy**	**AUC**	**Recall**	**Precision**	**F1**
Rf	Random forest classifier	0.7608	0.9063	0.7688	0.7715	0.7620
Lightgbm	Light gradient boosting machine	0.7552	0.8979	0.7646	0.7636	0.7651
Gbc	Gradient boosting classifier	0.7553	0.8938	0.7621	0.7586	0.7542
Et	Extra trees classifier	0.7533	0.9120	0.7598	0.7583	0.7529
Dt	Decision tree classifier	0.7514	0.8526	0.7596	0.7544	0.7513
Knn	K neighbors classifiers	0.6535	0.8299	0.6533	0.6622	0.6520
Lda	Linear discriminant analysis	0.6440	0.7938	0.6347	0.6472	0.6367
Ridge	Ridge classifier	0.6477	0.000	0.6361	0.6471	0.6289
Qda	Quadratic discriminant analysis	0.6422	0.7990	0.6318	0.6534	0.6180
Lr	Logistic regression	0.6270	0.7866	0.6211	0.6169	0.6110
Ada	Ada boost classifier	0.6159	0.7562	0.5995	0.6355	0.6101
Svm	SVM linear kernel	0.555	0.0000	0.5525	0.5849	0.5492
Nb	Naïve Bayes	0.5932	0.7757	0.5756	0.5853	0.5472
Dummy	Dummy classifier	0.3992	0.5000	0.3333	0.1594	0.2278

Shows the comparison between different ML algorithms using various performance metrics.

#### 2.3.3. Training of the hybrid model

**Figure 4:**
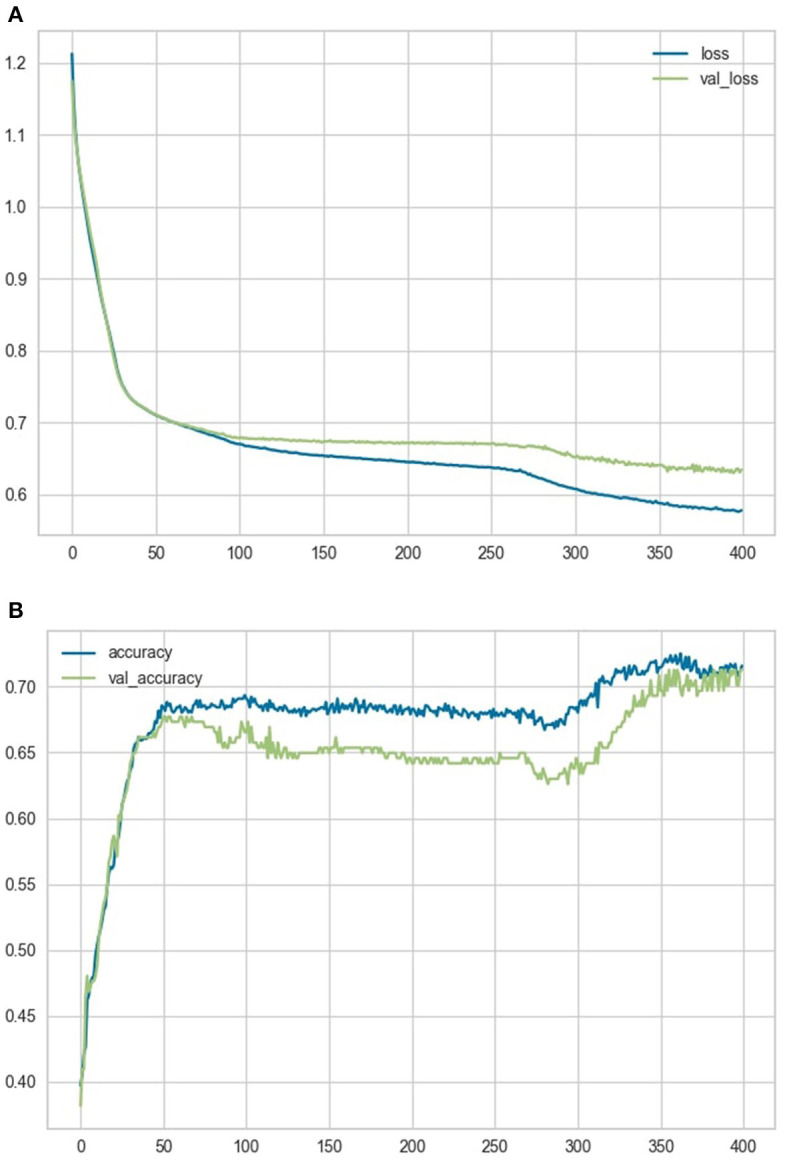
Loss and accuracy variables**. (A)** shows a comparison of the loss and validation loss variables over the course of training the model. **(B)** shows a comparison of the accuracy and validation accuracy variables over the course of training the model.

The ANN was trained on the training dataset for 400 epochs, while using the testing set simultaneously as a validation comparison to allow for overfitting assessment only. The loss and accuracy variables over the course of training the model are represented in Figures 4A, B.

The Random Forest (RF) Classifier was similarly trained on the same training dataset within the pycaret pipeline, 30% of the training dataset held out in the pipeline as validation data to prevent overfitting; a StratifiedKFold cross-validation was then conducted for a fixed number of 10 iterations to create a robust model. The model was then re-trained on the whole training data.

Afterwards, we merged these two classifiers into one final hybrid model (MaternalNET-RF). The hybrid model (MaternalNET-RF) utilized a maximum probability voting technique, this is by looking at the predictions (classes and probability values) of both the ANN and the RF classifier, it subsequently selects and outputs whichever class had the highest probability value regardless of difference in class predictions of both classifiers. The preference for this probabilistic approach stems from its demonstrated ability to offer simplicity and good interpretability, as similar maximum likelihood ensembles have shown in the past (Dembczyński et al., 2008). Additionally, since only 2 classifier units were employed in this multi-class prediction ensemble, the maximum probability voting technique capitalizes on their distinct natures, to the advantage that they will not commit the same errors (Leon et al., 2017). Thus, the result from the more confident classifier is selected as most correct. The model evaluation process included the utilization of a test set consisting of 254 data instances, representing a 25% hold-out.

## 3. Results

We evaluated the performance of our proposed hybrid model (MaternalNET-RF) on the following key metrics: Accuracy, Recall, Precision, F1 Score, and Area Under the Curve (AUC) Score (Bowers and Zhou, 2019; Powers, 2020). The findings from the StratifiedKFold cross-validation of the RF classifier are provided in Table 3, to showcase the utilization of the most robus model as an integral component within the hybrid model for testing. Our results subsequently showed that the ANN and RF classifier each had accuracy score of 0.7126 and 0.8858 respectively. This suggests that in the consideration of single models, traditional machine learning algorithms are better suited for this maternal health risk dataset, as there is no clear advantage of artificial neural networks in this case. An analysis of the detailed comparison of metrics between ANN, RF, and MaternalNET-RF provided in Table 4 reveals that the ANN performed below par in the classification of Mid Risk instances specifically, with a F1 score of 0.4500. This could be due to the small data size; thus, making proper generalization of the instance difficult. However, the ANN had excellent recall of the Low Risk instances with a recall score of 0.9000. The RF classifier was effective in classification across the categories but similarly struggled with the Mid Risk class. This is demonstrated by a Mid Risk F1 score of 0.8500, which is the lowest F1 score among the three risk categories.

**Table 3:** Stratified KFold cross-validation of the RF classifier.

**Fold**	**Accuracy**	**AUC**	**Recall**	**Precision**	**F1**
0	0.7778	0.9108	0.7836	0.8006	0.7818
1	0.7358	0.9030	0.7401	0.7509	0.7406
2	0.8113	0.9249	0.8200	0.8255	0.8130
3	0.7547	0.9012	0.7619	0.7579	0.7560
4	0.8113	0.9382	0.8175	0.8155	0.8122
5	0.8302	0.9343	0.8386	0.8352	0.8321
6	0.6792	0.8868	0.6772	0.6844	0.6815
7	0.8113	0.9118	0.8228	0.8157	0.8045
8	0.7547	0.9228	0.7698	0.7515	0.7517
9	0.6604	0.8413	0.6701	0.6769	0.6643
Mean	0.7627	0.9075	0.7702	0.7714	0.7638
Standard deviation	0.0540	0.0267	0.0565	0.0541	0.0537

Shows the complete 10-iteration StratifiedKFold cross-validation for the RF classifier.

**Table 4:** Performance comparison of ANN, RF classifier, and MaternalNET-RF.

**Model**	**Classes**	**Precision**	**Recall**	**F1 Score**	**AUC**	**Accuracy**
ANN	Low risk	0.6500	0.9000	0.7600	0.7784	0.7126
	Mid risk	0.6600	0.3500	0.4500		
	High risk	0.8700	0.8800	0.8800		
RF classifier	Low risk	0.9000	0.4100	0.9100	0.9133	0.8858
	Mid risk	0.8600	0.8500	0.8500		
	High risk	0.9000	0.9000	0.9000		
MaternalNET-RF	Low risk	0.9500	0.9400	0.9500	0.9622	0.9488
	Mid risk	0.9300	0.9400	0.9300		
	High risk	0.9700	0.9700	0.9700		

Shows an analysis of the detailed comparison of metrics between ANN, RF, and MaternalNET-RF.

Overall, the hybrid model (MaternalNET-RF) performed significantly better with an accuracy score of 0.9488. This increase in accuracy could be attributed to the fact that both models in the ensemble differ in the nature of their misclassifications, consequently drawing on each's distinctive nature; to the advantage of one another, to provide the most correct classification. The precision, recall and F1-score from our hybrid model ranged from 0.9300 to 0.9700.

**Figure 5:**
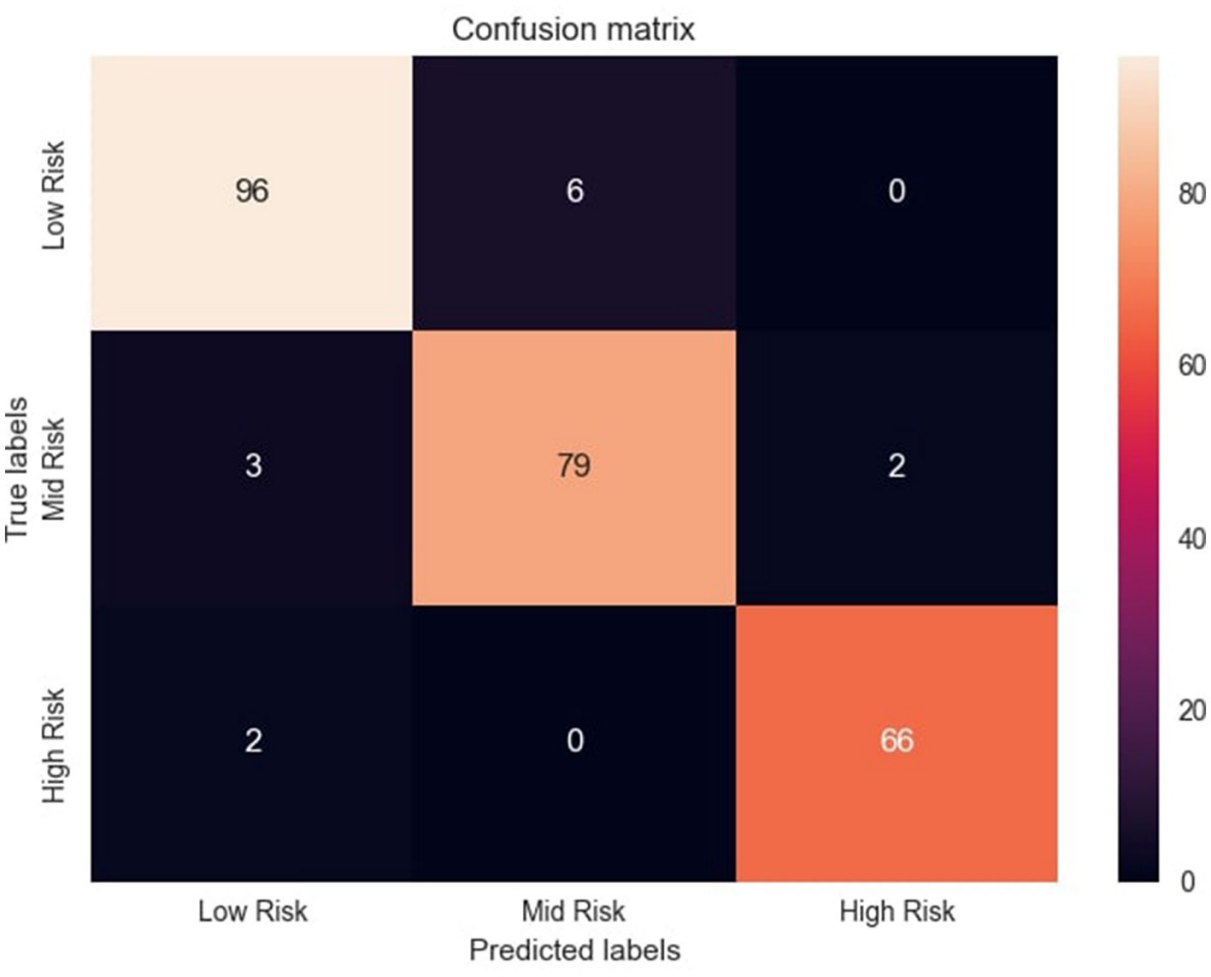
Confusion matrix of the model's predictions. Figure shows the confusion matrix with distribution of correct and incorrect predictions across risk categories.

The confusion matrix, illustrated in Figure 5, provides valuable insights into MaternalNET-RF's performance regarding classification of instances into Low Risk, Mid Risk, and High Risk. The analysis reveals that the model accurately predicted 96 instances as Low Risk, 79 instances as Mid Risk, and 66 instances as High Risk. Of particular significance is MaternalNET-RF's ability to avoid misclassifying any Low Risk instance as High Risk. This outcome is crucial, especially in real-world medical applications, as misclassifying a Low Risk instance as High Risk could have severe consequences. The model's ability to avoid such errors indicate its reliability and suitability for medical decision-making.

However, it should be noted that a few instances initially labeled as Low Risk were misclassified as Mid Risk. This misclassification might be attributed to the inherent non-linearity of the data and the subtle overlap between the characteristics of these two classes. Further insights and detailed information regarding correct classifications and misclassifications are provided in Figure 5. This allows for a more thorough examination of the model's performance, aiding in the understanding of its strengths and weaknesses in classifying instances into Low Risk, Mid Risk, and High Risk categories.

## 4. Discussion

The classification of maternal health risk is an important topic of discussion in the healthcare sector, particularly with regard to the wellbeing of women during pregnancy. Despite the availability of appropriate antenatal and postnatal care, some pregnant and nursing mothers choose not to utilize these services (Prasad et al., 2022). Various reasons have been identified for this behavior, including cultural, hereditary, social, economic, and political factors. In this regard, predictive AI tools such as MaternalNET-RF can efficiently identify high-risk pregnant women, allowing healthcare professionals to proactively intervene and provide personalized support. Maintaining maternal health remains crucial for the healthy development of the fetus during pregnancy. It is essential to closely monitor maternal health for any signs of disruption to normal bodily functions.

Common pregnancy complications such as hypertension or high blood pressure can restrict blood flow to the placenta, leading to inadequate nutrition for the fetus and increased risk of preterm labor and pre-eclampsia (Dimitriadis et al., 2023). Additionally, the weakened immune system of the mother during pregnancy makes her susceptible to various infections and conditions that could potentially harm the fetus (Amir et al., 2020). The implications of these findings highlight the importance of regular antenatal check-ups and timely management of any complications that may arise during pregnancy to ensure the wellbeing of both the mother and fetus. Undoubtedly, with proper pre-pregnancy, prenatal, and postpartum care, many of these complications can be prevented or treated. The growing popularity of emerging technologies such as Artificial Intelligence (A.I) and Internet of Things (IoT) technology has led to the development of smart IoT devices that can support maternal health.

Our findings indicate that the model was successful with an accuracy of 94.88%, a comparable result with other models built on the same data in past literature (Ahmed and Kashem, 2020; Raza et al., 2022). Ahmed and Kashem (Ahmed and Kashem, 2020) proposed a random forest model that achieved an impressive accuracy of 96% after cross-validation. However, further comparison could not be done as little is known about the model's architecture and hyperparameters. We would have loved to further compare other crucial performance metrics for evaluating the effectiveness of a multi-class prediction model; like precision, recall, and F1 score, but they were omitted.

Similarly, Raza, Siddiqui (Raza et al., 2022) proposed an architecture where a deep neural network and a decision tree are utilized for feature engineering and the data is subsequently predicted on several machine learning models, with Support Vector Machines (SVM) algorithm emerging as the best performing model, achieving an accuracy of 95% and an F1 score of 96%. However, the accuracy of the model increased to about 98% after data resampling was done using a data augmentation technique; Synthetic Minority Over-Sampling Technique (SMOTE). The use of data resampling techniques often improves a model's performance, but the limitation of this is that noise patterns may be introduced to the data, which may lead to model bias (Saglam and Cengiz, 2022). This difference in preprocessing techniques may be attributed to the difference in our results.

Notably, we provided detailed information about the model's architecture and hyperparameters, which can aid in reproducibility and generalization of our results. Additionally, we report precision, recall and F1 score in addition to accuracy, which provides a more comprehensive evaluation of our model's performance. Furthermore, we did not use any data resampling techniques, which can inadvertently lead to model bias.

Our hybrid model (MaternalNET-RF) has important implications for maternal health risk classification in clinical practice. By accurately identifying high-risk patients, healthcare providers can take preventative measures to reduce the likelihood of complications and improve maternal and fetal outcomes. This becomes even more important in low resource settings without specialists or skilled healthcare workers as it could help in predicting level of risk of pregnant women at antenatal. Invariably, the model can aid in resource allocation and efficient use of healthcare resources. Furthermore, due to the relative accessibility of the variables used in building the model, it could be quite possible that the model be incorporated into a web Application Point Interface (API) or an offline standalone application where the pregnant women can easily utilize from the comfort of their homes.

The high accuracy of the model suggests that an ensemble of deep learning and machine learning models has the potential to be useful tools in maternal health risk classification. However, our study is limited by the fact that the original author of the dataset did not furnish any explicit inclusion and exclusion criteria used in recruiting the participants. Consequently, variables such as blood group and genotype, history of chronic diseases, history of familial diseases, and infections that have the potential to influence maternal health outcomes could not be accounted for and thoroughly examined. Furthermore, the study was conducted on a small dataset, collected singly in the rural areas of Bangladesh. This might bias the model's ability to generalize, especially when it comes to its utilization on a global population or among Africans.

## 5. Recommendations

Future research should aim to collect more data and test the generalizability of our model on diverse populations. Various other suitable ensemble techniques can as well be explored, and the differences in metrics, and computational efficiency be compared. Moreover, there could also be the incorporation of other easily accessible variables not utilized in this study; such as packed cell volume, medication use, and vaccination history. Additionally, future research can leverage unstructured data such as text or images, in addition to structured variables used in this study. This allows more optimization and even better maternal health risk classification in pregnancy.

## 6. Conclusion

In conclusion, our study developed a novel deep hybrid model (MaternalNET-RF) for maternal health risk classification that achieved high accuracy and performed comparably to previous models. The model has important implications for clinical practice, and our findings suggest the ensemble technique of deep learning and machine learning models can be useful tools for maternal health risk classification. Future research is needed to confirm the generalizability of our findings and to optimize the use of structured and unstructured data in these models.

## Data availability statement

Publicly available datasets were analyzed in this study. This data can be found here: https://www.kaggle.com/datasets/csafrit2/maternal-health-risk-data.

## Author contributions

Conceptualization: TT and AB. Methodology: TT. Writing—original draft preparation and writing—review and editing: TT, AB, and K-u-RA. All authors have read and agreed to the published version of the manuscript.
